# The salt-inducible kinase KIN-29 regulates lifespan via the class II histone-deacetylase HDA-4

**DOI:** 10.17912/micropub.biology.000289

**Published:** 2020-08-09

**Authors:** Tatiana Nikooei, Aja McDonagh, Alexander M. van der Linden

**Affiliations:** 1 Department of Biology, University of Nevada, Reno, NV 89557

**Figure 1:**
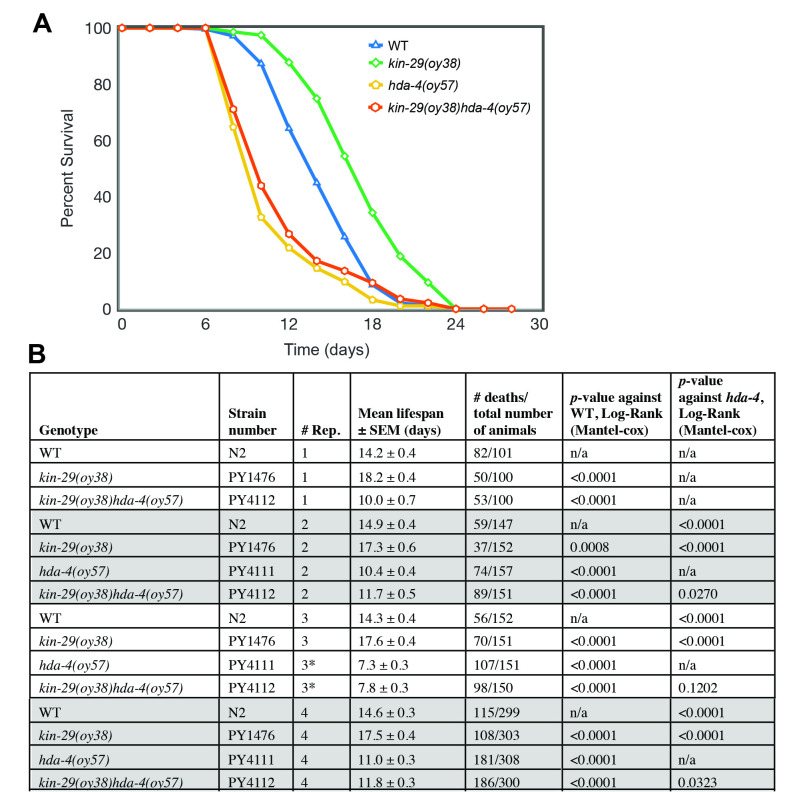
**Mutations in *hda-4* suppress the extended lifespan of *kin-29* mutants.** Lifespan was determined by assaying the percent survival of animals as a function of time. Scoring was performed every other day on NGM plates containing 5 μM FUDR and concentrated *E. coli* OP50 bacteria (see methods). Mean lifespans were with n>100 animals for each genotype (days ± SEM): *wild-type*, *kin-29(oy38)* and *hda-4(oy57)* single mutants, and *kin-29(oy38)hda-4(oy57)* double mutants. Data is shown from a single biological repeat (#4) with all strains examined in parallel. The mean lifespan of *hda-4* single and *kin-29hda-4* double mutants is significantly different from that of *wild-type* and *kin-29* single mutants, whereas the mean lifespan of *hda-4* single mutants is not statistically different from *kin-29hda-4* double mutants (log-rank test, p<0.01). * indicates animals fed at a higher food density. See table for data of each biological repeat and a summary of lifespan statistics.

## Description

*kin-29* encodes the *C. elegans* homolog of mammalian Salt-Inducible Kinases (SIKs). *kin-29* mutants are small, have increased propensity to develop into non-reproductive dauer larvae, have reduced chemoreceptor gene expression (Lanjuin and Sengupta, 2002; van der Linden *et al.*., 2007), have reduced cellular ATP despite increased fat stores, and show reduced sleep (Grubbs *et al.*, 2020). KIN-29 phosphorylates and inhibits the class II histone deacetylase 4 homolog HDA-4 to regulate gene expression in sensory neurons (van der Linden *et al.*, 2007). The longevity phenotype of *kin-29* mutants is suppressed by mutations in *daf-16* (Lanjuin and Sengupta, 2002), which encodes a forkhead box protein O (FOXO) transcription factor (Kenyon **et al.*,* 1993). These results indicated that the increased lifespan of *kin-29* mutants may be due to reduced insulin signaling.

To determine whether HDA-4 is required for the KIN-29 regulation of lifespan, we performed a survival analysis. As previously reported (Lanjuin and Sengupta, 2002), *kin-29(oy38)* mutants are long-lived. In contrast, *hda-4(oy57)* mutants are short lived (**Fig. 1**). The *kin-29hda-4* double mutant longevity phenotype was similar to the phenotype of *hda-4* single mutants (**Fig. 1**), suggesting that *hda-4* acts downstream of *kin-29* to regulate lifespan. Together with our previous findings (van der Linden **et al.*,* 2007), these genetic results suggest that KIN-29 may regulate lifespan via the action of HDA-4. Since SIKs can inhibit FOXO activity via HDAC4 to regulate gene expression (Mihaylova **et al.*,* 2011; Wang **et al.*,* 2011), it would be interesting in future studies to test the model that KIN-29/HDA-4 signaling converges on DAF-16/FOXO to modulate gene expression associated with longevity.

## Methods

All strains were maintained at 20°C for at least two generations before the lifespan assay. Lifespan assays were conducted at 20°C by placing age-synchronized L4 larvae of wild-type and the indicated genotypes on 6-cm diameter plates containing nematode growth media (NGM) plates and 5 μM 5’-fluoro-2’-deoxyuridine (FUDR) (Sigma) seeded with ~10^11^ *E. coli* OP50 bacterial cells per ml and 10-15 worms per plate. The number of dead hermaphrodites was scored every two days until all worms were dead, and the percentage of survival was calculated. Death was scored as a lack of movement after a nose touch. Animals that experienced ventral rupture, bagging or walling were censored from the analysis. ~100-300 animals from each strain were used for each biological replicate. Statistical analyses and survival curves were performed with the Online Application for Survival Analysis software (OASIS 2, https://sbi.postech.ac.kr/oasis2/ (Han **et al.*,* 2016). The significance of differences in mean lifespan was calculated using the Log-Rank test.

## Reagents

Strains used in this study are the wild-type strain N2 variety Bristol (Brenner, 1974), PY1476 *kin-29(oy38)*, PY4111 *hda-4(oy57)*, and PY4112 *kin-29(oy38)hda-4(oy57)* (Lanjuin and Sengupta, 2002; van der Linden **et al.*,* 2007). All strains are available at the CGC. The deletion/rearrangement in the *oy38* allele was confirmed by the polymerase chain reaction (PCR) and the single nucleotide change in *oy57* allele by sequencing of a PCR product.
